# Environmental and Demographic Determinants of Avian Influenza Viruses in Waterfowl across the Contiguous United States

**DOI:** 10.1371/journal.pone.0032729

**Published:** 2012-03-12

**Authors:** Matthew L. Farnsworth, Ryan S. Miller, Kerri Pedersen, Mark W. Lutman, Seth R. Swafford, Philip D. Riggs, Colleen T. Webb

**Affiliations:** 1 United States Department of Agriculture, Animal and Plant Health Inspection Service, Veterinary Service, Centers for Epidemiology and Animal Health, Fort Collins, Colorado, United States of America; 2 United States Department of Agriculture, Animal and Plant Health Inspection Service, Wildlife Services, National Wildlife Disease Program, Fort Collins, Colorado, United States of America; 3 Department of Biology, Colorado State University, Fort Collins, Colorado, United States of America; Erasmus Medical Center, Netherlands

## Abstract

Outbreaks of avian influenza in North American poultry have been linked to wild waterfowl. A first step towards understanding where and when avian influenza viruses might emerge from North American waterfowl is to identify environmental and demographic determinants of infection in their populations. Laboratory studies indicate water temperature as one determinant of environmental viral persistence and we explored this hypothesis at the landscape scale. We also hypothesized that the interval apparent prevalence in ducks within a local watershed during the overwintering season would influence infection probabilities during the following breeding season within the same local watershed. Using avian influenza virus surveillance data collected from 19,965 wild waterfowl across the contiguous United States between October 2006 and September 2009 We fit Logistic regression models relating the infection status of individual birds sampled on their breeding grounds to demographic characteristics, temperature, and interval apparent prevalence during the preceding overwintering season at the local watershed scale. We found strong support for sex, age, and species differences in the probability an individual duck tested positive for avian influenza virus. In addition, we found that for every seven days the local minimum temperature fell below zero, the chance an individual would test positive for avian influenza virus increased by 5.9 percent. We also found a twelve percent increase in the chance an individual would test positive during the breeding season for every ten percent increase in the interval apparent prevalence during the prior overwintering season. These results suggest that viral deposition in water and sub-freezing temperatures during the overwintering season may act as determinants of individual level infection risk during the subsequent breeding season. Our findings have implications for future surveillance activities in waterfowl and domestic poultry populations. Further study is needed to identify how these drivers might interact with other host-specific infection determinants, such as species phylogeny, immunological status, and behavioral characteristics.

## Introduction

Type A avian influenza virus (AIV) in wild waterfowl constitutes an important reservoir and source of infection for humans [1], [2], [3] and domestic poultry [4], [5]. In the United States AIV remains a threat to the domestic poultry industry [4], [5], [6] with estimated losses ranging from 5 to 212 million United States dollars [6], [7]. In North America there have been seven high-pathogenic outbreaks in poultry since 1924 with Losses from a single outbreak in 2007 estimated at 643 million Canadian dollars [8]. Although high pathogenic outbreaks of AIV in the United States have been rare, periodic outbreaks of low pathogenic AIV continue to occur and pose a threat due to its potential to mutate to the high pathogenic form of the virus. Wild waterfowl are well documented hosts for AIV [9], [10], [11]; however, identification of specific mechanisms structuring environmental infection risk across landscapes remains elusive. Experimental studies have established relationships between water temperature and chemistry (e.g., pH and salinity) and AIV persistence [12], [13]. At least one experimental study has documented the role of water as an indirect route of AIV transmission between individual waterfowl [14]. Other studies using simulation modeling [15], [16] have suggested that environmental reservoirs play a large role in maintenance of AIV in wild waterfowl. Currently there are few studies evaluating the results of laboratory-based experimental and simulation studies with field collected data [13]. Understanding determinants structuring the distribution of AIV in waterfowl is paramount to inform surveillance, monitoring, and outbreak response and management. Further, gaining an understanding of environmental drivers of infection is a necessary step towards national scale management of AIV spillover from wild waterfowl to poultry.

To mitigate evolving global risks associated with AIV, a United States interagency strategic plan was developed in 2006 for the early detection of (HPAI), specifically H5N1, to address the possibility that this virus might be introduced into the United States via wild birds during migration [17]. No highly pathogenic AIV have been identified in waterfowl within the United States; however, that surveillance effort has resulted in data on the presence or absence of type A influenza viruses within individual birds. The surveillance involved Federal and State Wildlife Agencies in all fifty States as well as University, Tribal, and State Department of Agriculture Cooperators. To date, the surveillance effort has resulted in the collection of more than 250,000 samples from wild migratory waterfowl. This represents the largest data collection effort regarding AIV in North American waterfowl.

Here we use a portion of the surveillance data to test hypotheses about the influence of local environmental conditions and past levels of infection on the probability an individual tests positive for AIV while accounting for demographic (e.g., species, age, and sex) and temporal patterns that are known correlates of AIV infection in wild waterfowl [10], [18], [19]. Based on studies suggesting a strong dependence of viral persistence on water temperature [12], [20], [21], [22], specifically greater persistence as temperature decreases, we hypothesized that environmental conditions (i.e., temperature) may influence local persistence of AIV and provide an epidemiological link between overwintering and breeding seasons. Because of the potential for AIV to persist in water for an extended period (e.g., up to 270 days) [23], [24], [25], we hypothesized that for a given site the proportion of AIV test-positive individuals across the overwintering period might be related to the probability of AIV infection at the individual bird level during the following breeding period within local watersheds. We also examined the influence of age and sex as it has been demonstrated that hatch-year birds and males are more likely to harbor AIV than older birds and females [9], [18], [19], [26], [27], [28]. In our statistical models, we wanted to control for these effects and determine if these patterns are consistent with our AIV surveillance data. As we move towards a more mechanistic understanding of the environmental conditions that give rise to variations in the geographic patterns of AIV, we will be increasingly enabled to improve surveillance and risk assessment for human and domestic poultry health. Our goals are to evaluate the significance of environmental, demographic, and temporal controls of AIV at a national level and to identify predictors of the distribution of AIV in wild waterfowl for informing disease management; specifically risk assessments and targeted surveillance.

## Methods

### Study site and data collection

Local wildlife biologists determined sampling locations based on success of waterfowl hunting, historical bird banding locations, and other factors. Specific study sites included State and Federal refuges, lakes, rivers, private hunting clubs, and other areas where waterfowl were legally hunted during open seasons or areas where live capture was easily facilitated, such as locations where historical waterfowl banding activities had occurred. Local expertise was used to further define the study area. Study sites were located throughout the contiguous United States and all birds were collected under the migratory bird scientific collecting permit for HPAI surveillance work (MB124992). No birds were lethally collected for the purpose of this study.

Two of the strategies identified in the Interagency Strategic Plan for Surveillance of AIV in Wild Waterfowl were hunter harvested birds and live wild birds. Specific information regarding the collection strategies is detailed in [17]. All samples were collected using standardized protocols and procedures [29]. Briefly, one cloacal and one oropharyngeal swab were collected from each bird using sterile Dacron-tipped swabs after which both swabs were combined in the same vial of transport media and left in the sample vial after collection. Samples were placed in cyrovials containing 3 mL of Brain Heart Infusion (BHI) transport media manufactured by Becton, Dickinson and Co., Sparks, Maryland, USA. Samples were shipped with ice packs within 24 hours of sample collection, whenever possible, and tested under a standardized protocol at one of 44 diagnostic laboratories that are part of the National Animal Health Laboratory Network. Laboratory handling protocols and testing procedures are described in detail elsewhere [30]. Because the surveillance effort was focused on first detection of H5 or H7 subtypes, no subtyping was conducted for any other Hemagglutinins.

### Data

For this analysis we used a subset of the AIV surveillance data in migratory wild birds across the contiguous United States, spanning from October 1, 2006 to September 30, 2009. Those data were further reduced to restrict our response variable for modeling of AIV matrix positive or negative to: 1) samples collected during the putative breeding season (April 1 to September 30); 2) birds that were sampled alive (i.e., not hunter shot); and 3) only dabbling ducks. We imposed these conditions for several reasons. First, we were interested in determining the influence of AIV infection during the wintering period on AIV status (test positive or negative) of individuals sampled in the subsequent breeding season. To examine this we used the interval apparent prevalence of AIV in birds sampled within a local watershed during the overwintering season (October 1 to March 31) as a predictor of the probability an individual would test positive during the following breeding season (April 1 to September 30) within the same local watershed. The interval apparent prevalence was defined for each watershed as the number of test positive individuals divided by the total number of individuals tested over a given length of time; in our case the six-month period from October 1 to March 31 prior to each breeding season. Thus, we only used data from the 137 local watersheds across the United States having both breeding and overwintering season data within the same biological year, April 1 to March 31. Second, we only considered birds that were sampled alive to reduce the likelihood of including early migrants in the analysis (e.g., American Green-winged Teal, *Anas carolinensis*) since samples from live birds were typically collected concurrent to traditional banding operations taking place on the breeding grounds. Finally, we restricted our analysis to the most common dabbling duck species (*Anatidae sp.*) in the surveillance data to reduce confounding with differing life history and habitat uses (e.g., Canada Geese) (Table 1). In addition, dabbling ducks are represented in much larger numbers than any other taxa in the surveillance data, which occurred because globally dabbling ducks have exhibited the highest AIV test-positive rates [10]. These restrictions resulted in a sample of 9996 birds from the breeding season and 9969 birds from the overwintering season. The response variable was the binary classification AIV test positive or negative during the breeding season for all models formulated for this study. Overwintering season AIV testing data was used to calculate one of the predictor variables described below.

**Table 1:** Species, number of individuals testing positive for influenza A virus matrix gene by rRT-PCR (Pos), number of individuals tested for AIV (Sampled), and point estimate of interval apparent prevalence (IAP) in breeding and overwintering seasons for each species across all three years of data used in this analysis.

	Breeding		Overwintering		IAP	
Species	Pos	Sampled	Pos	Sampled	Breeding	Overwintering
Mottled Duck	10	330	28	430	0.030	0.065
Gadwall	38	483	34	312	0.079	0.109
Northern Shoveler	5	61	11	63	0.082	0.175
Cinnamon Teal	7	74	6	28	0.095	0.214
Wood Duck	173	1815	124	3535	0.095	0.035
American Black Duck	24	145	10	294	0.166	0.034
American Wigeon	36	107	2	35	0.336	0.057
Blue-winged Teal	275	799	246	693	0.344	0.355
Northern Pintail	332	923	129	605	0.360	0.213
Mallard	1876	4918	1115	3833	0.381	0.291
Green-winged Teal	140	341	36	141	0.411	0.255

Predictor variables included a combination of intrinsic (i.e.; sex, age, and species) and extrinsic (i.e.; overwintering season temperature, interval apparent prevalence, and sampling month) variables. Age was classified as either hatch-year or after hatch-year birds. We included variables for species, age, and sex in the analysis to control for their putative effects and to account for as much demographic variability as the data allowed when estimating the effects of interval apparent prevalence and temperature. For a relatively small proportion of samples age and sex were unknown; thus, we included these as binary variables in our analysis with each bird coded as a “one” if age and sex were unknown and as a “zero” if age and sex were known. Because we wanted to control for individual- and species-level effects throughout the analysis, the variables age, age unknown, sex, sex unknown, and species always appeared together in models containing any of these effects. Additionally, a covariate for the month a sample was collected was included to account for the well documented pattern of increasing prevalence of AIV in the late summer and early fall months [9], [31].

Although the magnitude of these effects might be of interest from a landscape epidemiological perspective—and we report them here—they might also be viewed as nuisance variables included in the models to control for potential confounding with the two other variables of interest in this study. The first of those two variables was the interval apparent prevalence of AIV within local watersheds calculated for the previous overwintering period, which was used to capture the local influence of prior environmental deposition of AIV on breeding season infection probabilities. Local watersheds were derived from the United States Geological Survey's Hydrologic Unit Codes database [32]. The interval apparent prevalence of AIV within each hydrologic unit (i.e., local watershed) was calculated for each of the three overwintering seasons used in this analysis. Thus, for each year and watershed, the value of the interval apparent prevalence during the overwintering season was related to each bird tested during the subsequent breeding season within the same watershed. Finally, we included a temperature variable derived from National Oceanic and Atmospheric Administration weather station data [33]. For every bird sampled for AIV during the breeding season, we identified the nearest weather station (median distance = 14.7 kilometers) and calculated the total number of days having a minimum temperature below 0°C. Then, we adjusted for the difference in elevation between the weather station and the AIV sample location using the formula for environmental lapse rate:
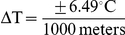
where 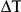 represents a change in temperature of 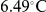 for every 1000 meters of elevation gained or lost between the weather station location and the AIV collection site. We included this variable as one hypothesis that might explain a portion of the spatial heterogeneity observed in the AIV surveillance data. This hypothesis was primarily based on published laboratory studies showing greater persistence of avian influenza viruses at colder temperatures, which in turn might result in a higher availability of AIV for infecting birds during the subsequent breeding season. All predictor variables were checked for excessive collinearity, which was sufficiently low not to preclude the use of all covariates in any combination in our candidate set of models.

### Formulation of competing models

We evaluated support for competing models portraying the relationship between the probability an individual bird sampled during the breeding season tested positive for AIV and the variables of interest: demographic (species, age, and sex), sampling month, interval apparent prevalence, and temperature. Dummy variable coding was used in which the estimated effect for each species was evaluated relative to mallards (*Anas platyrhynchos*). This coding was chosen because mallards were the most frequently sampled species in the data and they had one of the highest interval apparent prevalence values of any species; thus, all species effects were relative to the mallard effect. Given the ubiquitous distribution of mallards—and the fact that 44 percent of the overwintering and breeding season data used in this study were collected from that species—it is likely each of the local watersheds used in this study reflects a similar species composition. To check this assumption, we used a multiple pair-wise comparison test, the Marsculio procedure [34], to test for differences between the proportions of mallard versus all other species in each watershed when compared to all other 136 watersheds.

Our two primary hypotheses were:

1. a higher probability of testing positive during the breeding season would be associated with a higher observed interval apparent prevalence in the previous overwintering period at the local watershed scale; and
2. as the number of days in which the average temperature was reported below freezing during the six months prior to the breeding season increased, the probability an individual would test positive during the breeding season would increase.

These two phenomenological variables act as surrogates to capture geographic variability in the mechanisms of environmental deposition of AIV in feces and temperature mediated persistence during the overwintering season, respectively. All models containing the additive effects of age, age unknown, sex, sex unknown, and species were represented by the term “DEMO” in models where they appeared. The influence of sampling month was coded as “MONTH” in candidate models and interval apparent prevalence and temperature variables were coded as “IAP” and “TEMP”, respectively, in all models that included those terms.

We determined relative support in the data for candidate models to assess the influence of each variable, both alone and in the presence of the other variables, on the probability an individual bird tested positive for AIV during the breeding season. To assess the contribution made by each of the variables to predicting observed AIV status, we developed a suite of 16 candidate models that incorporated the variables in all possible additive combinations. All models assumed a binomial error structure and were fit using a generalized linear model with a logit link function. All models were of the form:
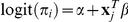
where 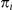 represented the probability that the *i*th individual tested positive for AIV, 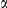 was the intercept representing the estimated background infection rate common to all waterfowl, 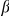 is an *m*×1 vector of regression coefficients corresponding to 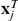, the transpose of the *m*×1 vector of covariates associated with the *i*th bird in the sample.

### Model selection

We used likelihood-based methods and information theoretics (Akaike's information criterion, AIC [35]) to estimate model parameters and quantify the strength of evidence for alternative models, respectively. Specifically, AIC was used to assess the relative information content of the models. Because model parameters were estimated based on data, there was some uncertainty the “best” model would emerge as superior if different data were used to compare alternatives. This uncertainty was quantified with Akaike weights, *w_r_* [35]. In the context of the analyses, we regarded normalized *w_r_* as “probabilities” that the estimated model *r* was the best Kullback-Leibler model for the data at hand, given the set of models considered [35]. The *w_r_* can be used to estimate the likelihood of the model, given the data, and in so doing offer a way to compare the relative weight of evidence for each model considered. Because AIC does not represent a goodness-of-fit metric, we developed Receiver Operating Characteristic (ROC) [36] curves and calculated Area Under the Curve (AUC) values to assess how well models fit the data. ROC curves, which originated in signal detection theory, plot the probability of detecting true signal (sensitivity) and false signal (1-specificity) for an entire range of cut points spanning the probability spectrum (i.e., 0 to 1), resulting in an AUC value lying between 0 and 1 for assessing model fit. All models contained only additive effects. Maximum likelihood estimates, confidence intervals on model parameters, and AIC values were obtained through logistic regression model fitting within the R computing environment [37].

### Model robustness

Although the choice of cutoff dates for representing breeding and wintering seasons correctly categorizes the biological activities of most birds in the data, there is some uncertainty around correctly classifying all birds in the sample with regard to being placed into the appropriate season. For example, migratory movements to and from breeding grounds in the spring and fall, respectively, differ by species, geographic location, and weather patterns in any given year. Because of this natural variability, we wanted to determine how robust biological inferences from our models were to variations in the time window considered for assigning birds to breeding and wintering seasons. To achieve this, the data was restructured such that each of the two original seasons were either delayed or advanced by one month, resulting in four new data sets, including new covariate values. The four time windows we considered reflected early (March 1 to September 30) and late (May 1 to September 30) starts to the breeding season, and early (September 1 to March 31) and late (November 1 to March 31) starts to the overwintering season. We refit the top model, based on AIC, to each of these new data sets and assessed how well the resulting parameter estimates reflected those from the top model fit to the data based on our original time window for breeding and overwintering seasons.

## Results

The Marsculio pair-wise comparisons test resulted in 3.7 percent of the local watersheds exhibiting a significant difference (p<0.05) in the proportions of each species relative to mallards. Thus, there appears to be little variation in species composition across the study area for those included in this analysis.

The results of our model selection exercise are shown in Table 2. Based on AIC, the global model (which contained an additive combination of all predictor variables) was the only model meriting consideration as the best approximating model in the candidate set. Because the Aikaike weight, *w_r_*, was effectively one (1), none of the other models were considered further and averaging parameter estimates across candidate models was unnecessary. Models were fitted in which we systematically omitted each of the variables making up the DEMO covariate, but with all other covariates included. However, those models all had AIC values that were much greater than the second best model in the original candidate set, which itself had an exceedingly small Akaike weight relative to the global model. Thus, retaining the DEMO composite variable in all of the candidate models was warranted.

**Table 2:** Candidate set of models used to identify the relative influence of covariates on the probability an individual bird sampled during the breeding season tested positive for Avian Influenza Virus (AIV).

Model	K†	Log-lik‡	ΔAIC§	wr*
DEMO + MONTH + DBZ + IAP	18	−5206.80	0	0.999
DEMO + ----------- + DBZ + IAP	17	−5225.70	35.81	1.68E-08
DEMO + MONTH + DBZ + ------	17	−5249.35	84.1	9.03E-19
DEMO + ----------- + DBZ + ------	16	−5266.82	116.04	6.35E-26
DEMO + MONTH + ------- + IAP	17	−5298.48	181.36	4.16E-40

Abbreviations are: DEMO = Demographic variables of age, age unknown, sex, sex unknown, and species; MONTH = sample month; DBZ = number of days temperature was below freezing during the six month overwintering season prior to each breeding season; and IAP = interval apparent prevalence of AIV within the local watershed during the six month overwintering period prior to each breeding season.*Notes:* Only the top five models are shown for clarity.

† Number of estimable parameters.

‡ Maximized value of the logarithm of the likelihood function.

§ Difference in AIC between a given model, *r*, and the model with the minimum AIC.

* Aikaike weight, *w_r_*, is the probability that the estimated model, *r*, was the best model given the data.

**Figure 1:**
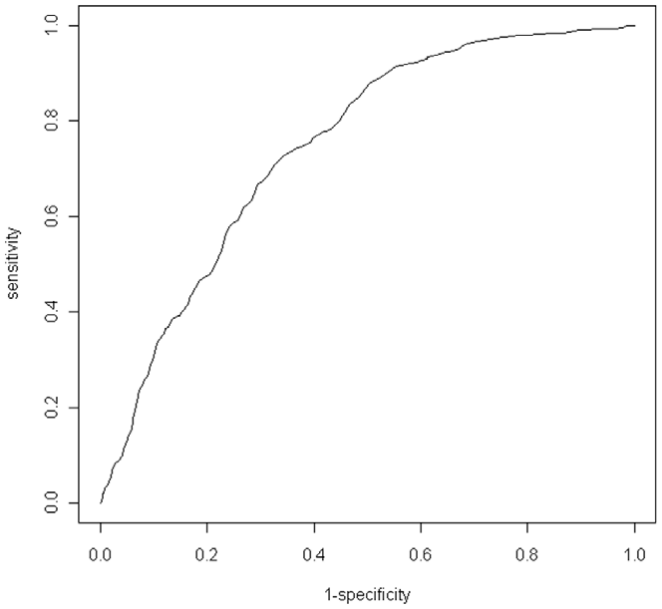
Receiver operating characteristics (ROC) curve for assessing goodness-of-fit for the top model selected from the candidate set. The area under the curve (AUC) is 0.76, suggesting a strong ability to discriminate between true and false signal and a good fit of the model to the data.

The trapezoidal rule was used to integrate the ROC curve associated with the global model (Figure 1) and calculated an AUC value of 0.76. AUC values between 0.70 and 0.80 are considered to have an acceptable level of discrimination between true and false signal [36]. This AUC value suggests that the global model fit the data well. Our assumption of binomially distributed errors was tested by refitting the same model but with a quasi-binomial error distribution, which allowed us to estimate the degree of overdispersion in the data. A perfect binomial process would have an overdispersion parameter equal to one. The point estimate for the overdispersion parameter using our avian influenza data was 1.02, suggesting that departures from the assumption of binomially distributed errors were negligible.

**Figure 2:**
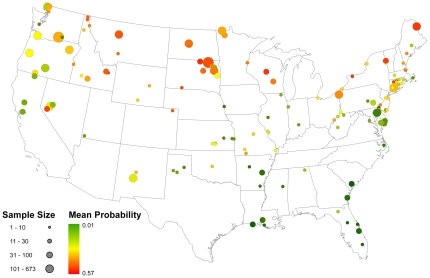
Top model estimate of average predicted probability that an individual bird sampled from local watersheds during the breeding season tests positive for avian influenza virus. The probability is an average across all three years of data for all waterfowl sampled within a given watershed. Note the strong latitudinal gradient with higher probabilities of testing positive in northern latitudes and decreasing probabilities in southern latitudes.

**Figure 3:**
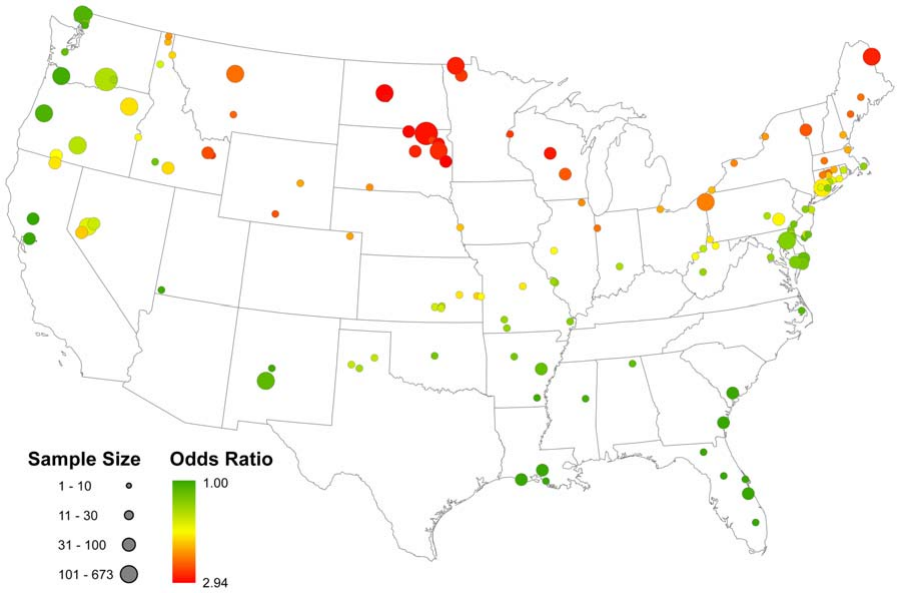
Map showing the odds ratio for the overwintering season temperature effect from the top model fit to the data. The sample size reflects the number of AIV samples collected within each of the 137 local watersheds used in this analysis and the colors reflect the mean odds ratio of testing positive for AIV, with red indicating that the odds of testing positive for AIV are nearly three times as likely than points colored dark green based on this variable.

**Figure 4:**
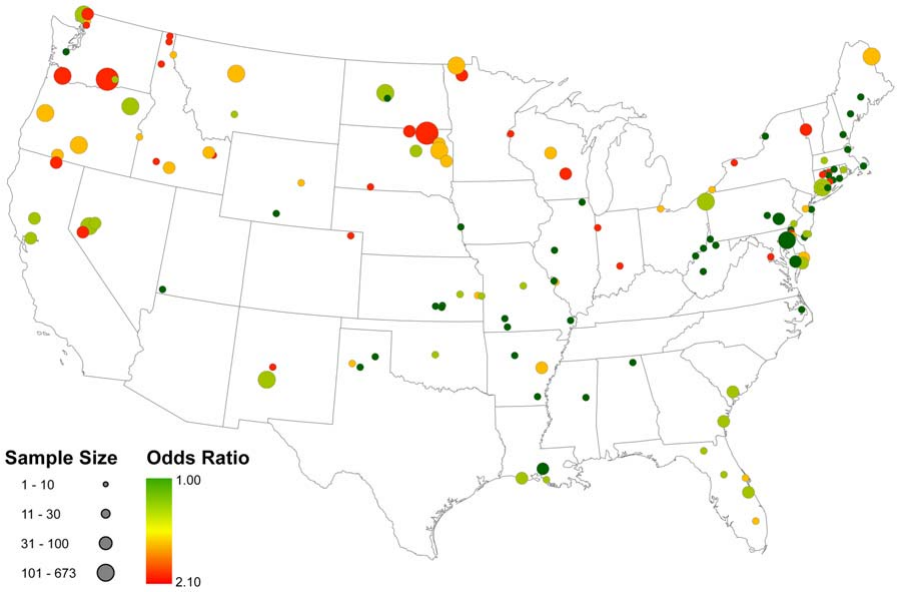
Map showing the odds ratio for the overwintering season interval apparent prevalence effect resulting from the top model fit to the data. The sample size reflects the number of AIV samples collected within each of the 137 local watersheds used in this analysis and the colors reflect the mean odds ratio of testing positive for AIV, with red indicating the odds of testing positive are more than twice that of points colored dark green based on this variable.

Figure 2 portrays the model-based probability that a randomly sampled bird from each watershed considered in this study would test positive for AIV during the breeding seasons considered in this study. The well-documented pattern of higher infection rates in northern latitudes is made apparent by this figure. Parameter estimates from the global model are shown in Table 3. All values are on the logit scale; however, exponentiation of these estimates provides an odds-ratio interpretation of their effect size. The cumulative number of days below 0°C during the overwintering season was a significant predictor of the probability an individual would test positive during the following breeding season (odds ratio = 1.008, 95% CI = 1.007, 1.009). For every seven days the local minimum temperature fell below zero, the chance an individual would test positive for AIV increased by 5.9 percent. Figure 3 maps this effect for all breeding season samples in the data set. Based on this variable, a bird sampled in North or South Dakota (northern States) during the breeding season would be expected to have nearly three times the odds of testing positive over a bird sampled on the Texas (southern State) gulf-coast during that same season. The final variable examined—interval apparent prevalence of AIV measured within local watersheds during the overwintering period prior to each breeding season—was also a significant predictor of the probability an individual would test positive during the following breeding season in that same local watershed (odds ratio = 3.13, 95%CI = 2.45, 3.98). This estimate translates into a 12 percent increase in the chances an individual will test positive during the breeding season for every 10 percent increase in the interval apparent prevalence during the prior overwintering period at the local watershed scale. Figure 4 depicts this relationship for all local watersheds collapsed across the three years of data covered by this study. These results suggest that the proportion of infected individuals during the overwintering season can be predictive of infection levels during the following breeding season over relatively small spatial scales.

**Table 3:** Maximum likelihood estimates for covariate parameters in the global model examining the relationship between the probability a bird tested positive during the breeding season (April 1 to September 30) and the variables shown.

Variable	MLE	2.5%	97.5%	p-value
Intercept	−3.418	−4.045	−2.799	<.001
DBZ	0.008	0.007	0.009	<.001
IAP	1.140	0.898	1.382	<.001
Hatch Year	0.695	0.595	0.796	<.001
Age Unknown	0.363	−0.012	0.723	.052
Sex	−0.156	−0.251	−0.061	.001
Sex Unknown	−1.341	−1.883	−0.851	<.001
Sampling Month	0.228	0.155	0.302	<.001
American Black Duck	−0.880	−1.360	−0.438	<.001
American Wigeon	−0.544	−0.968	−0.136	.010
Blue-winged Teal	−0.585	−0.758	−0.415	<.001
Northern Pintail	−0.305	−0.465	−0.147	<.001
Gadwall	−2.152	−2.508	−1.826	<.001
Northern Shoveler	−1.709	−2.791	−0.845	<.001
Green-winged Teal	−0.139	−0.382	0.102	0.259
Mottled Duck	−2.029	−2.736	−1.443	<.001
Wood Duck	−1.682	−1.856	−1.513	<.001
Cinnamon Teal	−1.433	−2.318	−0.708	<.001

DBZ (days below zero) is the cumulative number of days during the previous overwintering season that the mean temperature was less than zero and IAP is the interval apparent prevalence during the previous overwintering season. Hatch Year is the effect size relative to after-hatch-year birds, Age Unknown is the effect size relative to known age birds, Female is the effect size relative to males, Sex Unknown is the effect size relative to known sex birds, and all species effects are relative to the mallard. MLE is the maximum likelihood estimate of the parameter and 2.5 percent and 97.5 percent define the 95 percent confidence interval around the MLE based on profile likelihoods. All values are on the logit scale; however, exponentiation of these estimates provides an odds ratio interpretation of the effect size.

As expected, hatch-year birds had a greater probability of testing positive for AIV than after-hatch-year birds (odds ratio = 2.00, 95% CI = 1.81, 2.22) after accounting for species differences. Birds in which the age was unknown were also more likely to test positive for avian influenza (odds ratio = 1.44, 95% CI = 0.99, 2.06), with the confidence interval slightly overlapping 1 on its lower end. The variable female suggested differential infection probabilities, with females less likely to test positive than males (odds ratio = 0.856, 95% CI = 0.77, 0.94). When sex was unknown the results are even more skewed, with birds that could not be sexed being almost four times less likely to test positive (odds ratio = 0.26, 95% CI = 0.15, 0.42). The well-known observation of elevated infection rates in late summer and early fall months is confirmed in this analysis, with the odds of finding a test positive in the sample increasing as the breeding season progressed (odds ratio = 1.25, 95% CI = 1.17, 1.35). Species effects were also not surprising, given that all effects were relative to the mallard, which exhibited one of the highest proportions of test-positive individuals. The species with the lowest proportion of test-positive individuals during the breeding season, the gadwall (*Anas strepera*), was nearly ten times less likely to test positive during the breeding season than was the mallard (odds ratio = 0.12, 95% CI = 0.08, 0.16). Only one species was not significantly different from the mallard; a randomly sampled Green-winged Teal (*Anas carolinensis*) was nearly as likely to test positive as a mallard (odds ratio = 0.87, 95% CI = 0.68, 1.11).

The results from refitting the top model to the four new data sets reflecting variations in the assumed timing of breeding and wintering seasons are shown in Table 4 for several of the key variables we examined. Species effects are not shown for brevity; however, they exhibited very strong agreement with the original top model results. All effects shown in Table 4 are in very good agreement in terms of the sign, estimated values, and p-values, with the parameter estimates from the top model based on our original time window for the breeding and overwintering seasons. In particular, the number of days less than zero (DLZ), the sex effect, and the hatch-year effect show very strong consistency across all variations of assumed seasons. The interval apparent prevalence (IAP) and sample collection month show greater variability across the four new data sets, but are still consistent with parameter estimates from the top model based on the original asumed timing of breeding and overwintering seasons.

**Table 4:** Maximum Likelihood estimates (p-values) for key model parameters when AIV data are restructured such that the timing of breeding (April 1 to September 30) and overwintering (October 1 to March 31) seasons differs from that used to generate the parameter estimates shown in Table 3.

Season	DBZ	IAP	Sex	Hatch Year	Month
Original Dates	0.008 (<.001)	1.140 (<.001)	−0.156 (.001)	0.695 (<.001)	0.228 (<.001)
Early Spring	0.009 (<.001)	0.997 (<.001)	−0.154 (.002)	0.732 (<.001)	0.236 (<.001)
Late Spring	0.007 (<.001)	1.043 (<.001)	−0.156 (.002)	0.728 (<.001)	0.206 (<.001)
Early Winter	0.010 (<.001)	0.846 (<.001)	−0.141 (.028)	0.775 (<.001)	1.111 (<.001)
Late Winter	0.008 (<.001)	0.381 (.014)	−0.183 (.002)	0.827 (<.001)	0.170 (<.001)

Each of the two original seasons were delayed and advanced by one month, resulting in four new data sets to fit the top model. The four time windows reflected an early (March 1 to September 30) and a late (May 1 to September 30) breeding season, and an early (September 1 to March 31) and a late (November 1 to March 31) overwintering season. Key parameters include the number of days during the overwintering season having an average temperature less than zero degrees Celsius (DBZ), the interval apparent prevalence (IAP) during the overwintering season, the effect of being female (Sex), the age effect associated with hatch-year birds, and the month of sampling. Estimates of original model parameters and those from the four new time windows show strong agreement, suggesting that biological inferences from the top model using the original time window are robust to changes in the assumed timing of breeding and overwintering seasons. Species effects from the top model, not shown here, were also robust to changes in all four time windows and exhibited strong concordance with the estimated species effects from the top model based on the original time window.

## Discussion

To our knowledge, this study is the first examination of environmental drivers (e.g., temperature) of AIV using field data collected across the contiguous United States. It has been noted that a scarcity of field data exist for comparison to laboratory studies examining, among other aspects, environmental drivers of AIV infection [13]. The aim of this study was to address some of these knowledge gaps by characterizing determinants, including aspects of the environment, of AIV in wild waterfowl on their breeding grounds; a time when birds are much less mobile compared to the migratory season. By limiting our analysis to the breeding season and live sampled birds, we reduced much of the confounding in local spatial processes that would be introduced by combining AIV sample data from overwintering and breeding seasons into a single response variable. Although our results are consistent with previously reported patterns—specifically the increased proportion of infected hatch-year birds relative to after-hatch-year birds, an increase in the proportion of infected birds as the breeding season progressed, and males exhibiting higher test positive rates—we have nevertheless provided novel insight into potential landscape-level determinants of AIV in wild waterfowl across the contiguous United States.

In addition, we have shown that these results are highly robust to the assumed timing of the breeding and overwintering seasons, suggesting that our resulting biological inference is valid even in the face of natural variations in the timing of these two seasons. For example, our results should be applicable in years when the timing of breeding and overwintering migratory movements are either delayed or advanced relative to a “typical” breeding and overwintering season.

### Interval apparent prevalence

Based on the Marsculio test, it appears that across the 137 local watersheds examined in this study there is a strong consistency in the proportion of mallards in each watershed and that differential species composition between watersheds is an unlikely explanation for the overwintering season interval apparent prevalence effect observed in our top model.

At the local watershed scale there appears to be a linkage between overwintering and breeding seasons with respect to AIV infection. In locations where the proportion of birds testing positive was relatively high during the overwintering season the probability a bird tested positive during the following breeding season increased. This space-time linkage could be caused by multiple, possibly interacting, mechanisms. It may be that a similar composition of species uses the same watersheds for overwintering and breeding, which could lead to those species with overall lower or higher infection rates maintaining that pattern between seasons. Although we do not possess strain-level data, it may be more likely that those watersheds having a high interval apparent prevalence during the overwintering season represent areas where large quantities of virus are shed into water bodies used by foraging waterfowl, with persistence of a sufficient viral pool to facilitate transmission to birds using those same water bodies for breeding. The latter possibility suggests environmental persistence of AIV, at least within a given year, in which a reliable indicator of where a high proportion of infected individuals will be found during the breeding season (April 1 to September 30) is the interval apparent prevalence from the previous overwintering season (October 1 to March 31).

### Temperature

The effect of temperature on persistence of AIV in water has been well studied in laboratory environments [12], [13], [20], [21], [22]; however this is the first study to examine the influence of temperature on infection status in wild waterfowl of North America in a natural setting and across a large and heterogeneous landscape. Although the existence of a latitudinal gradient, with overall prevalence declining from north to south, has been noted previously [38] this is the first study we are aware of that examines a potential mechanism structuring that gradient. A primary factor, which has been cited in the past for this gradient, includes the presence of large numbers of hatch-year birds congregating on staging grounds in the northern States, particularly in the north-central portion of the United States. Although this is likely a dominant factor in observed prevalence levels in these areas, it is also likely that this host-virus system has evolved in a direction in which colder temperatures allow virus to persist longer, thereby seeding new infections upon arrival of large numbers of birds onto their breeding and pre-migration staging grounds. Because the temperature variable, in particular, was so invariant to changes in the assumed beginning and ending dates of the two seasons, we view this as a very robust predictor of breeding season interval apparent prevalence. Further field-based studies could examine this pattern at even finer spatial granularities, such as at the wildlife refuge level, to determine if this pattern is consistent across spatial scales.

### Species effects

**Figure 5:**
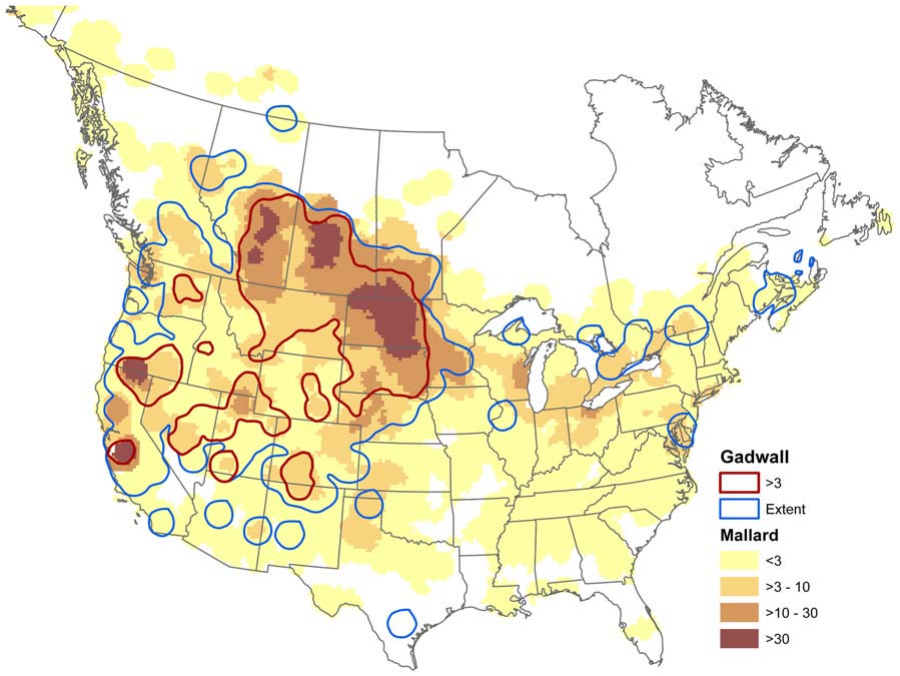
Map showing overlap in breeding relative abundance for mallard and gadwall species. Note that the geographic distribution of gadwall breeding locations is contained almost entirely by areas where mallard breed, with similar areas of high- and low-breeding concentrations across the contiguous United States. The mallard tested positive at some of the highest rates and the gadwall was near the lowest in proportion of AIV positive tests, suggesting geographic overlap alone does not explain variations in species prevalence patterns.

Variation in the interval apparent prevalence among species led predictably to a pattern of variability among species effects relative to the mallard (Table 1). The strength of the effect becomes increasingly negative as the interval apparent prevalence decreased among species. Given that the mallard has the greatest population size of all waterfowl in the contiguous United States, with an estimated 8.4 million individuals out of a total of 45.6 million waterfowl in 2011 [39], it is not surprising that this species exhibits one of the highest proportions of infected individuals among all those considered in this study. Avian influenza viral strains may exhibit differential reproductive capabilities between host species [40], [41], which can manifest itself in differential infection patterns among them [42]. Within this context, it is possible that a substantial portion of the AIV circulating in wild waterfowl environments has evolved to be more productive in mallards, which could potentially explain the high degree of variability in the interval apparent prevalence among the species examined in this study. For example, mallard and gadwall exhibited very different values; with gadwall testing positive at some of the lowest rates among all species and mallards testing positive at one of the highest rates. However, overlaying relative abundance maps of the breeding distributions for these two species shows that they are quite similar (Figure 5). Indeed, mallard and gadwall typically share the same breeding areas [43] and comingle extensively on the breeding grounds. It may be that viral strains circulating annually in waterfowl populations are predominately mallard adapted and that in this instance gadwall are weakly, or not at all, susceptible hosts for such strains. It is interesting to note that while mallards and American Green-winged teal exhibited similar AIV interval apparent prevalence values during the breeding season, they are much less likely to share local breeding areas than are mallard and gadwall [43]. Thus, the relationship between circulating AIV strains in any given year and host species competency remains a challenge to understand at the landscape scale.

### Sex Effect

Our analysis revealed a sex effect, something which has been observed previously in Alaska [18] and Canada [19], but has not been reported elsewhere in North America, with males having a higher probability of testing positive for AIV. Although we do not have data that allow us to confirm a specific mechanism, several hypotheses regarding this effect have been suggested. The effect could be due to a combination of physiological and space use differences between male and female dabbling ducks. Male ducks typically have elevated testosterone levels during the breeding season [44], [45], [46], which has been shown to decrease immune function [47], [48]. Conversely, elevated levels of estrogen, most notably in females, have been shown to increase immune function [49], [50], [51]. It is possible that these differential hormone patterns are linked to differences in AIV infection probabilities between the sexes. Further, males are more likely to switch mates between breeding seasons, thus potentially using a greater number of breeding sites over their lifetime than females [52]. This could lead to exposure of males to a greater number of AIV strains than females, resulting in a higher infection probability as males encounter a greater number of strains throughout their life to which they are, at least partially, immunologically naïve. Finally, female mallards—along with many other dabbling duck species—suffer higher mortality rates on their breeding grounds [53]. If females infected with AIV are more likely to suffer mortality through, for example, increased predation risk or other mortality factors linked to disease, this could lead to the patterns observed in these data due to censoring of AIV infected females. Each of these potential explanations remains an open question requiring further study to determine the mechanisms behind the observed sex effect. Data from other studies should be used to the extent possible to either confirm, or refute, our findings in terms of the differential probability of testing positive between the sexes.

### Implications for poultry health

Spillover from wild waterfowl has been implicated in outbreaks of AIV in domestic poultry [31], [54], [55], [56]. In the United States this has been of particular concern in the Great Lakes region where the turkey industry has experienced production losses resulting from AIV [31], [55], [57] and in the New England region where the live bird marketing system has repeatedly experienced outbreaks of AIV [58], [59], [60]. Furthermore the presence of AIV in wild waterfowl has been linked to increased transmission efficiency among sympatric populations of domestic poultry [61]. The increasing evidence that wild waterfowl directly influences the incidence of AIV in domestic poultry populations suggests a need for developing tools to aid in identifying regions at risk and support mitigation of transmission events and optimized surveillance.

Our results indicate an increased probability of AIV infection in waterfowl in regions that have historically experienced AIV in domestic poultry – Great Lakes and New England regions. While our analysis did not explicitly address the relationship between AIV infection in waterfowl and poultry, it does suggest a potential relationship. Should a highly pathogenic zoonotic strain emerge in North America from wild waterfowl or poultry, or from a reassortant from the two populations, these regions may be looked to as a potential source given the sympatric waterfowl and poultry populations, locally high waterfowl AIV prevalence, high occurrence of low biosecurity backyard poultry operations [62], [63], live bird markets [64], [65], and small commercial poultry operations [66], [67].

Enhancing surveillance activities in regions with higher probabilities of AIV infection in waterfowl may also yield long-term benefits for early detection of novel AIV strains. The ability to target locations where a high incidence of AIV is likely to occur during the breeding season, which also culminates with the annual peak in AIV prevalence in waterfowl, has implications for prevention and mitigation of disease in poultry. A current challenge to addressing this issue is optimizing surveillance systems to improve early detection of AIV, particularly novel strains, and identifying regions that would receive the largest benefits from establishment of risk mitigations to prevent transmission. Adjuncts to more traditional surveillance approaches may be warranted, such as adjusting the level of surveillance based on monitoring results during the overwintering season. Using the overwintering season AIV status for a given location or region may serve as an indicator of increased AIV transmission during the following breeding period which might, in turn, increase potential risk for spillover to poultry. In addition, identification of these areas before the breeding season may allow for implementation of risk-based mitigations (e.g., reduced contact between free-range poultry and waterfowl) which can serve as a valuable method of reducing potential spillover and subsequent outbreaks and production losses.

### Future directions

This is the first analysis we are aware of that examines the role of environmental features and infection history as determinants of AIV within North America from a landscape perspective. We have shown how local-scale epidemiological history and temperature observed in the overwintering season can influence the probability of infection at the individual level during the following breeding season; however, further mechanistic studies are needed to determine the relative role of these and other environmental drivers. The combination of a large proportion of infected individuals, and an increase in the length of below freezing temperatures during the overwintering season, suggests that the proportion of individuals shedding virus—and temperature mediated environmental persistence between the two periods—may be a coupled mechanism influencing the spatial epidemiology of this host-parasite system. Our results suggest that future breeding season surveillance efforts could be made more efficient by weighting surveillance activities towards locations where a high interval apparent prevalence of AIV was observed during the previous overwintering season, and where temperatures remain below freezing for relatively extended periods. Given the scarcity of landscape epidemiological work in this system, greater attention is needed to disentangle the mechanisms driving infection probabilities at individual and population levels. Although work has been conducted examining the differential ability of species adapted strains to infect other species [42], there remains a lack of information linking host phylogenies to differential susceptibility and viral replication. Linking this information with host life history traits would further identify patterns of differential species risks for exposure and infection. Such information could provide valuable insight into the observed variability in infection rates among species. Disentangling the role of environmental persistence, differential behavior, and immunological capacity—as it relates to infection rates at individual and population levels—will require large-scale observational studies and experiments which have not been attempted to date; but which could provide valuable information for improving surveillance systems for early detection of HPAI strains circulating silently in North American wild migratory waterfowl.
